# The Influence of Small Quantities of Oxygen in the Structure, Microstructure, Hardness, Elasticity Modulus and Cytocompatibility of Ti-Zr Alloys for Dental Applications

**DOI:** 10.3390/ma7010542

**Published:** 2014-01-20

**Authors:** Fábio B. Vicente, Diego R. N. Correa, Tatiani A. G. Donato, Victor E. Arana-Chavez, Marília A. R. Buzalaf, Carlos R. Grandini

**Affiliations:** 1 UNESP—Univ. Estadual Paulista, Laboratório de Anelasticidade e Biomateriais, 17.033-360, Bauru, SP, Brazil; E-Mails: fabiobv@fc.unesp.br (F.B.V); diegornc@fc.unesp.br (D.R.N.C.); 2 USP—Universidade de São Paulo, Faculdade de Odontologia, Departamento de Biomateriais e Biologia Oral, 05.508-900, São Paulo, SP, Brazil; E-Mails: tatianidonato@gmail.com (T.A.G.D.); vearana@usp.br (V.E.A.-C.); 3 USP—Universidade de São Paulo, Faculdade de Odontologia, Departamento de Ciências Biológicas, 17.012-901, Bauru, SP, Brazil; E-Mail: mbuzalaf@fob.usp.br

**Keywords:** Ti alloys, interstitials, microstructure, mechanical properties, biocompatibility

## Abstract

The mechanical properties of Ti alloys are changed significantly with the addition of interstitial elements, such as oxygen. Because oxygen is a strong stabilizer of the α phase and has an effect on hardening in a solid solution, it has aroused great interest in the biomedical area. In this paper, Ti-Zr alloys were subjected to a doping process with small amounts of oxygen. The influence of interstitial oxygen in the structure, microstructure and some selected mechanical properties of interest for use as biomaterial and biocompatibility of the alloys were analyzed. The results showed that in the range of 0.02 wt% to 0.04 wt%, oxygen has no influence on the structure, microstructure or biocompatibility of the studied alloys, but causes hardening of the alloys, increasing the values of the microhardness and causing variation in the elasticity modulus values.

## 1. Introduction

Currently, there is a need to find new materials for orthopedic and dental uses, mainly in relation to their mechanical properties, considering that the alloys used commercially in prostheses have different mechanical properties compared to human bone. To meet this need, research is looking for metals and alloys with excellent biocompatibility, chemically satisfactory passivity and good durability post implantation [1].

Ti and its alloys have abundant applications in the area of biomaterials. Its main properties are excellent corrosion resistance, relatively low elasticity modulus, high specific strength and good biocompatibility [1,2]. The development of new Ti alloys aims to obtain materials with favorable properties for use in the human body. The new alloys are being developed with the addition of Mo, Nb, Zr and Ta, because these elements do not cause cytotoxicity [3,4].

Zr is considered a neutral element in a solid solution with Ti, because it does not affect the β-transus temperature of the allotropic transformation. It presents high solubility in both crystalline phases of Ti, and it forms solid solutions in several concentrations [5,6]. As a substitutional element, it causes the hardening of the alloy, increases the corrosion resistance and improves biocompatibility [7]. The addition of the element in the formation of Ti alloys can even provide a decrease in the temperature of martensitic transformation and the melting point of the material [8,9].

Among the studied binary alloys, the Ti-Zr system presents advantageous properties for its application as a biomaterial, such as good tensile strength [10], low density and good biocompatibility. Zr has chemical properties similar to Ti, and the formation of solid solutions occurs with a certain ease. This set of properties is frequently studied and applied in orthodontics [10–13].

The effect of interstitial elements on the microstructure and mechanical properties of Ti alloys has awakened great interest in several works in the literature [14,15]. Oxygen is a strong Ti α phase stabilizer, showing a greater effect than Al in phase stabilization [15,16]. Earlier studies report that oxygen can cause hardening of the alloy, as well as influence the superelasticity and shape memory effect in some Ti alloys. The formation of martensite is another effect evidenced in some studies, though little understood [17–19].

In this paper, the influence of interstitial oxygen in the structure, microstructure, select mechanical properties and biocompatibility of Ti-*x*Zr alloys (*x* = 5, 10 and 15 wt%) was analyzed. X-ray diffraction and optical microscopy measurements were conducted for the analysis of the structure and microstructure. The selected mechanical properties were obtained using Vickers microhardness tests and dynamic elasticity modulus.

### 2. Results and Discussion

The chemical composition of the produced alloys is presented in Table 1. The results of the chemical analysis indicated that the material produced has good quality, with the main elements in concentrations close to the stoichiometry and with negligible quantities of impurities [7]. Table 2 presents the amount of oxygen present in all studied samples.

**Figure 1.**
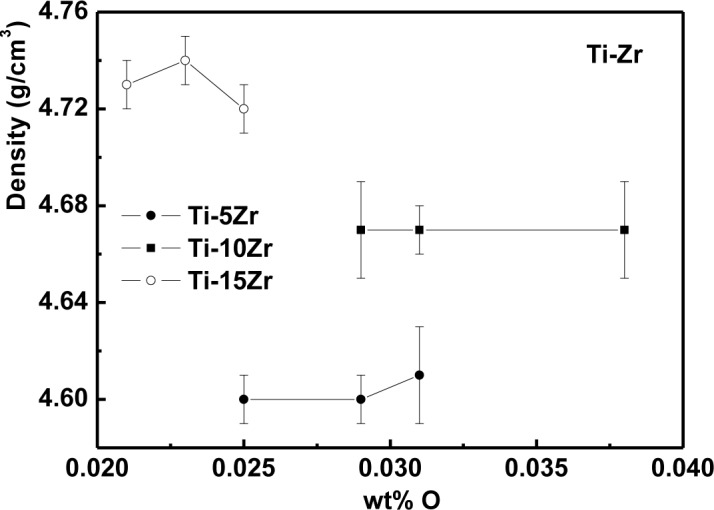
Density as a function of oxygen content in Ti-Zr alloys.

The density values as a function of oxygen content are presented in Figure 1 for Ti-5Zr, Ti-10Zr and Ti-15Zr. It can be observed that the density values did not suffer significative variations of the interstitial element, once the oxygen variation in the sample contributes negligibly to the density variation. The density values were also quite close to the theoretical value of the alloys: 4.56, 4.61 and 4.69 g/cm^3^, for 5, 10 and 15 wt% of Zr, respectively [20].

**Figure 2.**
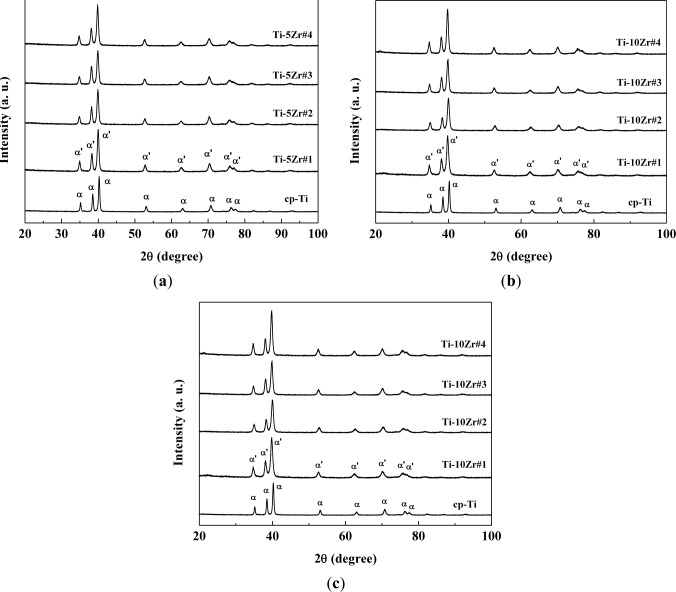
X-ray diffractograms for (**a**) Ti-5wt%Zr (Ti-5Zr); (**b**) Ti-10wt%Zr (Ti-10Zr) and (**c**) Ti-15wt%Zr (Ti-15Zr) alloys, in all conditions studied.

Figure 2 shows the X-ray diffractograms of the studied samples compared with commercially pure titanium. It can be observed that in all the studied samples, the hexagonal compact crystalline structure is present [6,8,10–13,21], and there is no significant change of crystalline structure with the variation of the oxygen concentration. The oxygen concentration is too low to observe significative structural changes. The peaks found were related to the martensitic α’ phase, which has a distorted hexagonal compact structure. It can be clearly observed that oxygen concentrations do not significantly alter the peaks position in the diffractograms. As oxygen is an α phase stabilizer and Zr is a neutral element, it was expected that the material does not exhibit phase changes [6,14]. The diffractograms follow the results of the literature for Ti-Zr alloys, where the peaks of the retained β phase were found only at concentrations above 50 wt% of Zr [5,6,22]. There is a small displacement of the diffraction peaks for smaller angles, which can be the result of the increase of the lattice parameters of the crystalline structure. This dilation is mainly related to the addition of Zr in solid solution, because of the great difference in their atomic radius (1.58 Å) compared with Ti (1.47 Å) [18,20]. To verify this assumption, the diffractograms were analyzed using the Rietveld method, and the parameters obtained are shown in Table 3. The fit residual (Rwp) and χ^2^ parameters are factors that indicate the quality of refinement [23]. In so-called perfect conditions, the Rwp parameter must be between 0% and 10%, while χ^2^ must be exactly one. However, because the Rietveld method takes into account various experimental factors (such as powder size and equipment failure), the values found are satisfactory [23–25].

It can be verified that in all treatments, the α’ phase was prevalent, including when the sample was quickly cooled in the case of hot swaging. The presence of lubricating and coolant fluid in the swaging wheels significantly reduces the temperature of the samples. Thus, it can be concluded that the aggressive treatments to the material were not sufficient to stabilize the β phase, but martensitic formation may have occurred for these alloys, because the peaks of the α’ martensitic phase are in the same positions as the peaks of the α phase [23–26]. The substitutional Zr increases the lattice parameter, which was expected, because the atomic radius of Zr is expected to be higher than that of Ti, making the unit cell larger.

**Figure 3.**
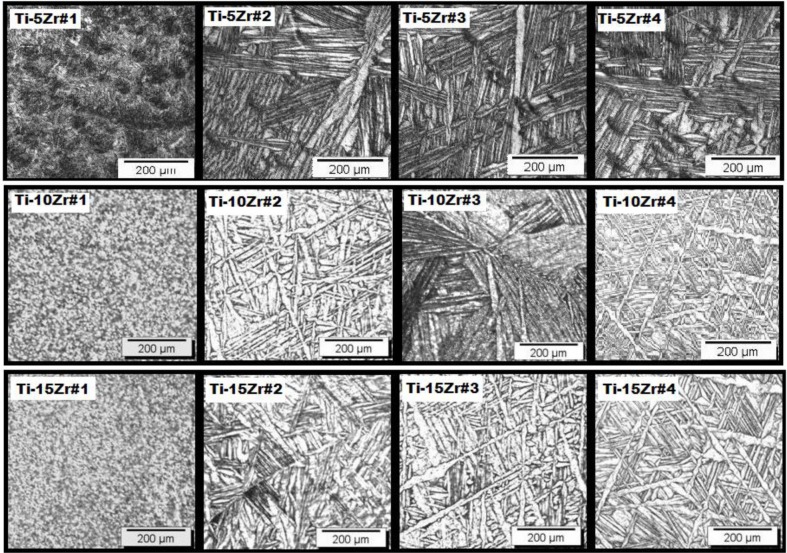
Optical micrographs for Ti-Zr alloys.

The micrographs of the alloys after the doping steps are shown in Figure 3 and show an acicular-shaped microstructure, typical of the titanium martensitic α’ phase. The addition of Zr increases the formation of this phase, because it is not done as a homogenizing heat treatment [1,6]. It is observed in Figure 3 that the martensitic formation became denser (Widmanstätten pattern) after the heat treatment, indicating the removal of internal stresses, due to the swaging process. Even after the heat treatment, the basket weave-type structure is present throughout the sample, characteristic of Ti alloys of the hexagonal system. The formation of the martensite occurs due to the addition of Zr, because the element has a strong effect on decreasing the start of the martensitic transformation temperature [6,18]. The variation in the interstitial oxygen concentration did not cause changes in the microstructure, as shown earlier by the results of X-ray diffraction. The presence of interstitial oxygen facilitates the formation of the martensitic phase, because the presence of this element in the material distorts the crystalline lattice, causing crystallite microstrain [27–30].

**Figure 4.**
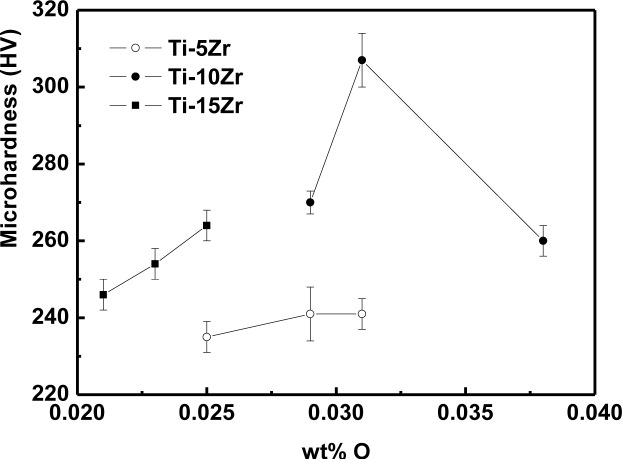
Microhardness as a function of oxygen content for Ti-Zr alloys.

The variation of microhardness values as a function of oxygen concentration is shown in Figure 4. The microhardness of the alloys presented an increasing tendency toward the interstitial oxygen concentration. The addition of oxygen as an interstitial element increases the difficulty of the dislocation motion, causing a hardening of the material. In the hcp crystalline structure, the interstitial elements are not free to move, and this truncation of the dislocations reduces the atomic mobility of the interstitial region [17], increasing the hardness of the material. Silva *et al*. [19] obtained the same behavior in binary Ti-Nb alloys. The highest value of the microhardness among the studied alloys for Ti-10Zr with 0.038% of oxygen was expected, but probably a great part of the oxygen was trapped in the grain boundaries, not contributing to increasing of the hardness of the alloy. All conditions presented microhardness values higher than cp-Ti (187 ± 4 HV), due to the solid solution hardening caused by interstitial oxygen and substitutional Zr.

**Figure 5.**
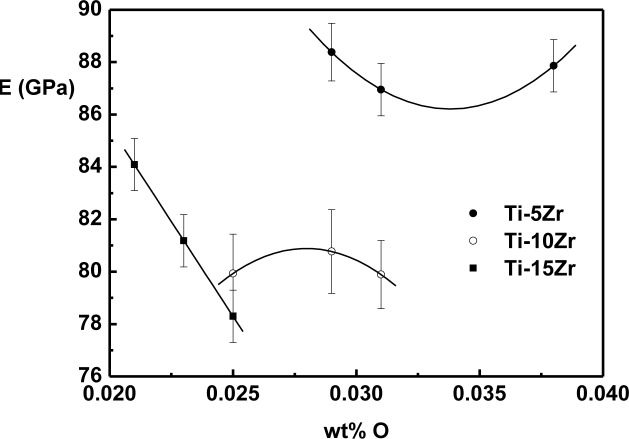
Elasticity modulus as a function of oxygen content for Ti-Zr alloys.

The effect of oxygen on the elasticity modulus of Ti-Zr alloys is shown in Figure 5. The values were obtained at 37 °C, which is approximately human body temperature.

Different behaviors of the elasticity modulus as a function of oxygen were observed in the binary system Ti-Zr. For alloys with 5 wt% of Zr, there is a decrease of the modulus until around 0.035 wt% of oxygen, and after this value, there is a growing trend. For the alloy with 10 wt% of Zr, modulus value growth can be observed, reaching a maximum value around 0.027 wt% of oxygen, and after this value, there is a tendency of a reduction in the value of the modulus. In the case of the alloy with 15 wt% of Zr, a near-linear decrease of the elasticity modulus with the interstitial oxygen concentration is observed. The reduction in the value of the elasticity modulus can be related to the weakening of atomic bond forces due to interstitial oxygen [9,31]. Theoretically, in Ti alloys in which the predominant phase is α or α’, oxygen atoms are not free to move, and this interstitial immobility generates a decrease in the elasticity modulus [32,33]. However, the effect of interstitial oxygen in the elasticity modulus is not yet fully understood, distinct behaviors being found in Ti-Mo [33,34], Ti-Nb [19] and Ti-Ta [35]. In all cases, the elasticity modulus is lower than cp-Ti.

**Figure 6.**
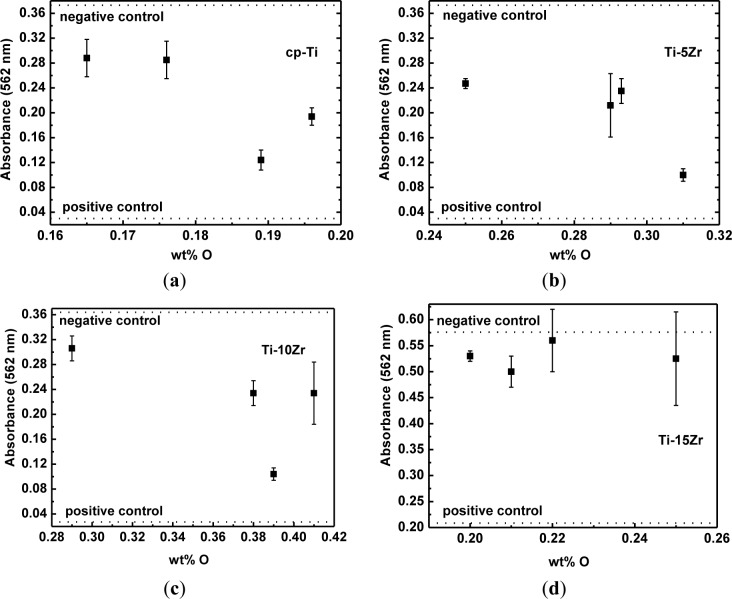
Direct cytotoxicity tests as a function of the oxygen concentration for (**a**) cp-Ti; (**b**) Ti-5Zr; (**c**) Ti-10Zr and (**d**) Ti-15Zr alloys.

Figure 6 shows the results of the measurements of absorbance (direct cytotoxic tests) for cp-Ti and the studied alloys as a function of oxygen concentration, as well as the negative and positive cytotoxicity control. It can be observed that the number of viable cells is nearly the same as that of cp-Ti. The number of viable cells cultured on the Ti-5Zr alloy remains constant, but decreased for oxygen content around 0.031 wt%. For the Ti-10Zr alloy, a slight decrease with the oxygen content increase was observed. For the alloy containing 0.039 wt% of oxygen, a more significant decrease in the viable cells were observed. In both situations, there are conditions with a greater quantity of the β phase. For the Ti-15Zr alloy, no statistically significant variations in the number of viable cells cultured on the samples are observed, with the increase in the oxygen content. This will be the subject of new studies. It is observed that in all tests, the results are above the positive control and below the negative control and showed similar results to those observed in the tests with cp-Ti. Thus, it can be said that the inclusion of oxygen did not negatively influence the biocompatibility of the alloys [36].

The development of new titanium alloys is intended to produce a material with properties more suitable for application in the human body. In the case of dental implants, a high mechanical resistance is required, due to the need for hard mechanical work in the region [14,18]. For implants used in joint, hip and knee replacements, an elasticity modulus near human bone (around 30 GPa) is very important to ensure successful implants and patient comfort [4,37]. The increase in hardness values, as well as the decrease of the elasticity modulus with the oxygen concentration, makes the alloys a great potential for use as a biomaterial.

## 3. Experimental Section

The samples used are Ti-5Zr, Ti-10Zr and Ti-15Zr alloys, produced from cylindrical commercially pure titanium bars (99.7% purity, Sigma-Aldrich Inc., St. Louis, MI, USA) and zirconium foil (99.6% purity, Sigma-Aldrich Inc.). The melting of the metals was completed in an arc furnace with a water-cooled copper crucible and argon atmosphere. The samples were melted at least five times to ensure a good homogeneity. The analysis of the chemical composition has been performed using an optical emission spectrometer with induced plasma.

The produced ingots were swaged, with a 10% reduction of diameter by step, and the resulting bars were cut using a precision saw into cylindrical bars 4.0 mm in diameter and 40.0 mm in length. A homogenizing heat treatment was later performed to relieve internal stresses caused by swaging (Condition #1). The treatment was carried out under a vacuum of 10^−7^ mbar, with a heating rate of 10 °C/min and a holding temperature of 1000 °C by 24 h, followed by slow cooling. The gas doping process was conducted in a quartz tube under a vacuum of 10^−7^ mbar, a heating rate of 10 °C/min and a holding temperature of 900 °C for 2.0 h and quick cooling with water. The partial pressures of oxygen introduced into the quartz tube were 10^−2^ (Condition #2), 10^−1^ (Condition #3) and 100 (Condition #4) Torr. To obtain the amount of oxygen in the material, gas analysis was performed by thermoconductivity difference using LECO model TC-400 equipment. The density was obtained using the Archimedes’ method with an analytical balance.

The analysis of the structure and microstructure of the produced alloys was performed by X-ray diffraction and optical microscopy, respectively. The diffraction pattern was obtained in a Rigaku D/Max 2100/PC diffractometer, using the powder method, with CuKα radiation (λ = 1.544 Å) in a scan of 2°/min in the range of 10° to 100°. Microstructural analysis was performed using an Olympus BX51M optical microscope.

The selected mechanical properties were evaluated by microhardness and elasticity modulus measurements. Vickers microhardness measurements were obtained in a Shimadzu microdurometer, HMV-2 model, with a load of 200 g (1.961 N) for 60 s. The elasticity modulus was obtained in a torsion pendulum, with an oscillation frequency of 30 Hz, under a vacuum of 10^−6^ mbar with a heating rate of 1 K/min and a temperature range of 250 to 350 K [38,39].

The MC3T3-E1 cell lineages (preosteoblastic lineage obtained from the calvaria of *Mus musculus*; ATCC, Rockville, MD, USA) were used in the biological tests. The cell viability (MTT test) was measured using an absorbance reading [40]. The MTT analysis was done with an experimental time of 72 h. Polystyrene (culture plate) was used as the negative control, while a solution of α-MEM + 10% of fetal bovine serum (FBS) + 1% phenol was the positive control for cytotoxicity. After 72 h, MTT (5 mg/mL) was added to each well, and the plate was incubated at 37 °C for 3 h. In this sequence, the medium was removed and replated with 100 μL DMSO to dissolve the formazan crystals. The product was quantified spectrophotometrically by measuring the absorbance at 562 nm using a microplate reader.

## 4. Conclusions

The prepared samples have good uniformity and stoichiometry. The gas doping process was effective, and it was possible to observe that the oxygen concentrations did not cause significant changes in the microstructure of the alloys. The formation of the martensite α’ phase was caused by the addition of Zr in a solid solution.

The values of microhardness presented an increase according to the variation in oxygen concentration, showing a hardening of the material. For the elasticity modulus, it was concluded that all the samples studied have a lower elasticity modulus than cp-Ti and that the introduction of oxygen has an influence on the values of the modulus.

Cytocompatibility tests conducted on the samples showed, in an initial analysis, that the material does not present cytotoxic effects. The fact that heat treatments and oxygen doping did not negatively interfere with the cytocompatibility shows that the studied alloys are promising for biomedical use.

## Figures and Tables

**Table 1.** Chemical composition (in wt%) of the Ti-Zr produced alloys.

Sample	Zr	Fe	Cr	Ni	Al	Ti
Ti-5Zr	4.89	0.03	0.01	0.009	0.001	balance
Ti-10Zr	9.76	0.03	0.01	0.008	0.006	balance
Ti-15Zr	15.60	0.04	0.01	0.005	0.009	balance

**Table 2.** Oxygen content of the produced samples (wt%).

Sample	#1	#2	#3	#4
Ti-5Zr	0.029 ± 0.001	0.031 ± 0.001	0.025 ± 0.001	0.029 ± 0.001
Ti-10Zr	0.029 ± 0.001	0.029 ± 0.001	0.031 ± 0.001	0.038 ± 0.001
Ti-15Zr	0.020 ± 0.001	0.021 ± 0.001	0.023 ± 0.001	0.025 ± 0.001

**Table 3.** Parameters obtained using the Rietveld method.

Sample	Rwp (%)	χ^2^	*a* (Å)	*c* (Å)	α’ phase (%)	β phase (%)
Ti-5Zr #1	11.50	1.897	2.9618 (2)	4.6964 (4)	99.98	0.02
Ti-5Zr #2	13.33	2.211	2.9616 (3)	4.6965 (4)	99.97	0.03
Ti-5Zr #3	13.49	2.399	2.9616 (3)	4.6977 (5)	99.98	0.02
Ti-5Zr #4	11.59	1.879	2.9614 (3)	4.6969 (5)	99.99	0.01
Ti-10Zr #1	8.34	1.419	2.9697 (4)	4.7054 (5)	99.99	0.01
Ti-10Zr #2	11.33	1.564	2.9701 (3)	4.7099 (5)	99.99	0.01
Ti-10Zr #3	12.81	1.985	2.9704 (3)	4.7111 (5)	99.99	0.01
Ti-10Zr #4	9.74	1.635	2.9715 (3)	4.7118 (5)	99.97	0.03
Ti-15Zr #1	9.02	2.107	2.9801 (2)	4.7223 (5)	99.99	0.01
Ti-15Zr #2	7.57	1.808	2.9806 (2)	4.7215 (4)	99.99	0.01
Ti-15Zr #3	8.71	2.345	2.9801 (3)	4.7236 (4)	99.97	0.03
Ti-15Zr #4	8.88	2.828	2.9888 (3)	4.7214 (6)	99.39	0.06
